# Supplementary material for: A Critical View of the Application of the APEX Software (Aqueous Photochemistry of Environmentally-Occurring Xenobiotics) to Predict Photoreaction Kinetics in Surface Freshwaters
Source: Molecules. 2019 Dec 18;25(1):9. doi: 10.3390/molecules25010009 (PMC7017383; doi:10.3390/molecules25010009)
Supplement: Supplementary file 1 [file molecules-25-00009-s001.zip › 3.2.4_gcc-4.4.0.zip!/3.2.4_gcc-4.4.0/3.2.4_gcc-4.4.0/doc/octave/html/interpreter/Orthogonal-Collocation.html]

Orthogonal Collocation - Untitled


Previous: Functions of Multiple Variables,
Up: Numerical Integration


---

### 22.2 Orthogonal Collocation

— Loadable Function: [r, amat, bmat, q] = **colloc** (n, "left", "right")  
> Compute derivative and integral weight matrices for orthogonal
> collocation using the subroutines given in J. Villadsen and
> M. L. Michelsen, Solution of Differential Equation Models by
> Polynomial Approximation.

Here is an example of using `colloc` to generate weight matrices
for solving the second order differential equation
u' - alpha \* u” = 0 with the boundary conditions
u(0) = 0 and u(1) = 1.

First, we can generate the weight matrices for n points (including
the endpoints of the interval), and incorporate the boundary conditions
in the right hand side (for a specific value of
alpha).

```
     n = 7;
     alpha = 0.1;
     [r, a, b] = colloc (n-2, "left", "right");
     at = a(2:n-1,2:n-1);
     bt = b(2:n-1,2:n-1);
     rhs = alpha * b(2:n-1,n) - a(2:n-1,n);
```

Then the solution at the roots r is

```
     u = [ 0; (at - alpha * bt) \ rhs; 1]
          => [ 0.00; 0.004; 0.01 0.00; 0.12; 0.62; 1.00 ]
```
